# Senescent T Cells: The Silent Culprit in Acute Myeloid Leukemia Progression?

**DOI:** 10.3390/ijms252312550

**Published:** 2024-11-22

**Authors:** Xiaolan Zhang, Lingbo Liu

**Affiliations:** Institute of Hematology, Union Hospital, Tongji Medical College, Huazhong University of Science and Technology, Wuhan 430022, China; zxl18737469816@126.com

**Keywords:** T cell senescence, acute myeloid leukemia (AML), tumor microenvironment (TME), therapeutic intervention

## Abstract

Malignant tumors can evade immune surveillance and elimination through multiple mechanisms, with the induction of immune cell dysfunction serving as a crucial strategy. Mounting evidence indicates that T cell senescence constitutes the primary mechanism underlying T cell dysfunction in acute myeloid leukemia (AML) and represents one of the potential causes of immunotherapy failure. AML usually progresses rapidly and is highly susceptible to drug resistance, thereby resulting in recurrence and patient mortality. Hence, disrupting the immune interface within the bone marrow microenvironment of AML has emerged as a critical objective for synergistically enhancing tumor immunotherapy. In this review, we summarize the general characteristics, distinctive phenotypes, and regulatory signaling networks of senescent T cells and highlight their potential clinical significance in the bone marrow microenvironment of AML. Additionally, we discuss potential therapeutic strategies for alleviating and reversing T cell senescence.

## 1. Introduction

Acute myeloid leukemia (AML) is a malignant clonal disorder featuring widespread heterogeneity, primary chemotherapy resistance, and a high recurrence rate [1]. It is characterized by the proliferation of myeloid blasts with impeded differentiation and the suppression of normal hematopoiesis. It clinically manifests as infection, bleeding, anemia, and the infiltration of extramedullary tissue [2]. Considerable alterations have transpired in the treatment of AML in recent years, yet more than two-thirds of patients still experience a tragic consequence of drug resistance and relapse. Emerging immunotherapies are envisaged to eliminate chemotherapy-resistant clones and achieve long-term disease control, encompassing treatment approaches such as immune checkpoint inhibitors (ICIs), chimeric antigen receptor (CAR)-T cell therapy, and monoclonal antibodies (mAbs) [3]. AML cells play an active role in the immune editing process, representing a potential target for immunotherapy [4]. As the most potent immune cells capable of eliminating tumor cells, T cell dysfunction, to a certain extent, can adversely impact the efficacy of immunotherapy. Senescence might be the principal mechanism underlying CD8^+^ T cell dysfunction in AML, accounting for the unsatisfactory responses to immune checkpoint blockade (ICB) therapy observed thus far [5]. Senescence takes place in different physiological and pathological processes, such as aging or cancer. The age-associated immune system dysfunction is referred to as immunosenescence, which is characterized by thymic involution, metabolic dysregulation, naïve/memory cell ratio imbalance, and epigenetic alterations. It is commonly associated with infections, age-related disease onset, and tumors. In contrast, cellular senescence, characterized by cell cycle arrest and diverse forms of cellular damage, is regarded as the cause of tissue and organismal senescence. During the process of immune senescence, exhausted, aged, and senescent T cells can be observed [6]. Senescent T cells may be the major driving force of immunosenescence.

A growing body of evidence indicates that T cell senescence is prevalent in suppressive tumor microenvironments (TMEs). Significant accumulations of senescent CD8^+^ T cells have been identified in the tumor-infiltrating lymphocytes (TILs) associated with diverse types of cancers, which sustain and magnify the suppressive tumor microenvironment by potently inhibiting the proliferation of reactive T cells, including those in AML, multiple myeloma (MM), melanoma, and breast cancer [7,8,9,10]. Nevertheless, the interaction mechanism between the AML TME and senescent T cells remains incompletely understood, and knowledge of the consecutive dynamic immune landscape of the leukemia TME is still lacking [11]. Notably, the clinical influence of senescent T cells on AML has garnered extensive attention in recent years, encompassing the development of prognostic indicators and the evaluation of treatment response. The identification of immune dysfunction and immune-related risk factors is essential for a full comprehension of the genesis and progression of AML and the prediction of immunotherapy responses, potentially opening novel avenues for immunotherapy. Here, we summarize the general characteristics and formation mechanisms of T cell senescence and focus mainly on the evidence and progression of T cell senescence in the AML TME. Additionally, treatment strategies that may restore and reverse the senescence of T cells are proposed.

## 2. Characteristics of Senescent T Cells

**Figure 1:**
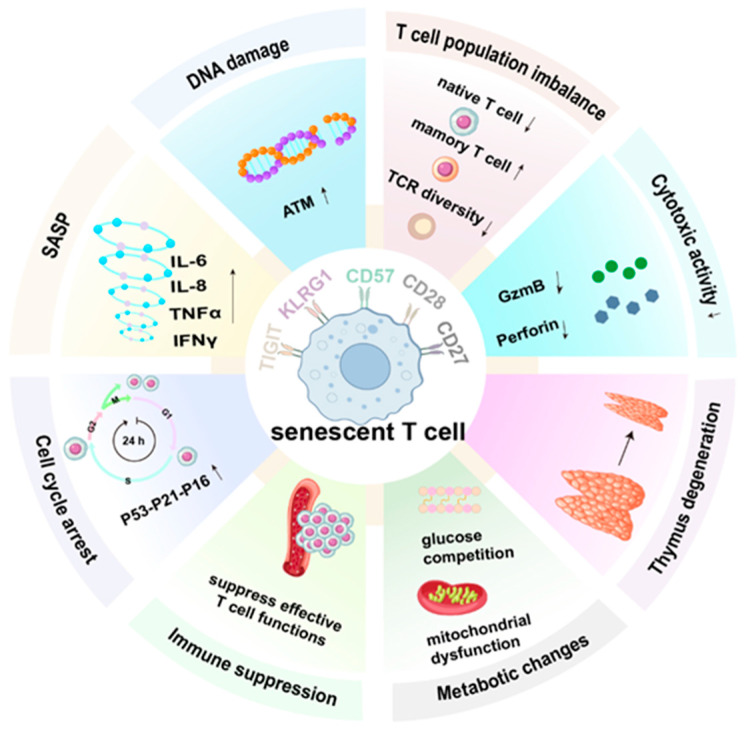
T cell senescence. T cell senescence can manifest as the senescence of individual cells and dysregulation of the cell population. In addition to the common characteristics of general cell senescence, senescent T cells can be characterized by the loss of surface CD27 and CD28 and the upregulation of terminal differentiation markers. Functionally, senescent T cells demonstrate impaired self-killing and immunosuppressive effects. ↑ represents the activation or upregulation; ↓ represents suppression or downregulation.

In order to accurately assess the immune environment, T cells can be quantified into eight continuous states: quiescence, regulating, proliferation, helper, cytotoxicity, progenitor exhaustion, terminal exhaustion, and senescence. Under the pressures of aging, chronic antigenic stimulation, autoimmune disorders, and malignant tumors, T cells enter a phase of arrested growth and proliferation and are termed “senescent T cells” [12] (Figure 1). Thymic atrophy might also be a potential driving force in the aging landscape of T cells, resulting in a reduction in immunity and tissue repair capacity [13]. Comprehending the traits of T cell senescence can facilitate a more comprehensive understanding of disease progression from an immune perspective and enable effective interference. Similar to the aging of common cells, senescent T cells grow larger and flatter, exhibit elevated expression of senescence-associated β-galactosidase (SA-β-Gal), possess shortened telomeres due to repetitive replication of the telomeres, lose telomerase activity, and have increased DNA damage [14,15]. The upregulation of *p53*, *p21*, and *p16*, coupled with the downregulation of *cycle-dependent kinase2* (*Cdk2*), *Cdk6*, and *cyclin D3* (*CCND3*), gives rise to irreversible cell cycle arrest and the loss of the proliferative capacity in senescent T cells [10,16,17,18]. Senescent T cells can regulate the tumor microenvironment and perform intercellular communication through the senescence-associated secretory phenotype (SASP) and maintain metabolic activity, producing substantial amounts of inflammatory cytokines such as interleukin-2(IL-2), IL-6, IL-8, tumor necrosis factor (TNF), and interferon-gamma (IFN-γ), as well as inhibitory cytokines such as IL-10 and transforming growth factor beta (TGF-β) [19,20]. Unlike normal cells, senescent T cells are characterized by the loss of the T cell receptor (TCR) costimulatory receptors CD27 and CD28, the upregulation of terminal differentiation markers such as killer cell lectin-like receptor G1 (KLRG1), and the re-expression of CD45RA [21,22,23,24]. As a result, the deficiency of CD27 and CD28 can lead to defects in cytotoxicity and influence the progression of AML [25]. CD57 constitutes another terminal differentiation marker for identifying human senescent T cells and has been verified to be linked with severe proliferative impairment [26]. CD57^+^T cells are frequently associated with the loss of CD28. The coinhibitory receptor T cell immunoreceptor with Ig and ITIM domains (TIGIT) serves as an efficient marker of immune senescence. Recent research has indicated that the coexpression of TIGIT and the transcription factor Helios refines the definition of immune senescent CD8^+^ T cells, thereby challenging the dogma that the late differentiated stage acts as a surrogate for T cell immune senescence [27,28]. 

Functionally, on one hand, senescent T cells exhibit impaired killing capacity as a consequence of the loss or dysfunction of perforin and granzyme. On the other hand, they can transform into immunosuppressive cells, potently suppressing the proliferation and effector functions of other immune cells, thereby attaining the amplification of immune suppression [20,29,30]. In addition to aging characteristics at the individual-cell level, T cell senescence can also manifest as imbalances in cell populations. The quantity of naïve T cell subpopulations decreases, whereas that of memory T cells increases, resulting in an imbalance in the proportion of the T cell repertoire [31,32]. Nevertheless, other studies have indicated that CD4^+^ T cells derived from elderly individuals are prone to differentiate into effector-like cells rather than into memory cells. This alteration is associated with the equilibrium among Helios, a member of the IKAROS transcription factor family, IL-2Rα, and signal transducer and activator of transcription 5 (STAT5) [33]. The diversity of TCRs decreases with increasing age and long-term antigen exposure. Moreover, TCR-mediated signal transduction is attenuated, which might be associated with the expression or activity of TCR downstream signaling molecules [21,34].

## 3. T Cell Senescence in the AML Microenvironment

Even though basic genomics predominantly delineates the stage, degree of risk, and probability of clinical response to conventional therapeutics in AML, the evaluation of immune dysfunction in the AML microenvironment can act as a more comprehensive adjunct to prognostic and risk assessment.

### 3.1. Evidence of T Cell Senescence in the AML Microenvironment

**Figure 2:**
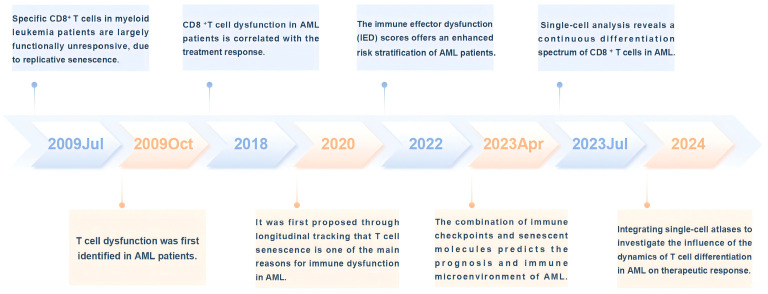
Timeline of events related to the progression of T cell senescence in AML.

Despite considerable advancements in therapeutic approaches for AML, a substantial proportion of patients ultimately experience relapse and refractory conditions, with a 5-year overall survival (OS) rate of only 9.4% in patients over 65 years old. Notably, a minor fraction of leukemia cells (subsequently referred to as leukemia stem cells (LSCs)) can regenerate leukemia. LSC is regarded as the principal initiator of the occurrence, persistence, and recurrence of AML [35,36]. As immune senescence progresses, immune dysfunction in AML patients might also be associated with therapy-resistant relapse. However, comprehensive analyses of immunological characteristics during the development and treatment of AML are lacking, and little is known regarding the relationships between immune status and disease occurrence or treatment response. With the continuous development of computational tools, scientists have utilized single-cell and spatial multiomics technologies to continuously explore the TME, map the immune landscape, and construct models of tumor immune evasion. We summarize the research progress on T cell senescence in AML and present the results in Figure 2.

Initially, Rifca Le Dieu et al. reported that the T cells of AML patients presented abnormal phenotypes and genotypes and formed defective immune synapses. This was the first explicit report of T cell dysfunction in AML, which could lead to a failure of the immune response against leukemia cells [37]. Furthermore, patients who achieved remission following hematopoietic stem cell transplantation presented a considerable number of functionally unresponsive CD28^−^CD57^+^CD8^+^ T cells with shorter telomeres, which was consistent with cell senescence [38]. Since then, researchers have gradually acknowledged the key role of T cell aging in the progression of AML, with numerous studies focused on how it affects the treatment response of patients and predicts prognosis. Knaus HA et al. found that AML blasts can directly modulate the viability, expansion, cosignal transduction, and senescence marker expression of CD8^+^ T cells in vitro and that the dysfunction of T cells is associated with the treatment response [8]. Prospective studies and multiparameter flow cytometry revealed that T cell senescence and exhaustion, along with impaired natural killer (NK) and γδ T cell functions, constitute the main factors contributing to immune dysfunction in AML, representing the first longitudinal assessment of the immune status in AML [39]. Another landmark scRNA-seq study revealed that senescent-like bone marrow CD8^+^ T cells were compromised in the elimination of autologous AML blast cells and that their proportion was negatively correlated with OS. Researchers have proposed new immune effector dysfunction (IED) characteristics and reported that IED scores are associated with adverse risk molecular aberrations, stemness, and poor outcomes, which serve as independent OS prediction criteria, enhance AML risk stratification and might facilitate the provision of personalized immunotherapy to patients [7]. This observation revealed a previously underestimated connection between the AML immune microenvironment and clinical risk. In addition to the advancements in single-cell technology, the most recent technological progress in analyzing phenotypes and transcriptional states has revealed substantial heterogeneity in T cell states within tumors among patients and between patients. Notably, this heterogeneity renders it imperative to understand the relationship between individual T cell states and treatment responses [40,41]. Jing-Min Yang et al. developed a novel T cell state identifier, designated “TCellSI,” which is capable of assessing diverse T cell states mentioned above on the basis of transcriptome data. The T cell state score (TCSS) can be further utilized for prognosis and the prediction of immunotherapy response [42]. Another investigation employed high-dimensional flow cytometry and single-cell transcriptomics to elucidate the dynamics of CD8^+^ T cell differentiation within the AML microenvironment. They discovered that early memory CD8^+^ T cells were correlated with the treatment response and manifested a bifurcation into two disparate terminal states. One state was enriched with activation markers, whereas the other expressed NK-like and senescent markers. The clonal differentiation trajectory toward CD8^+^ senescence is also a marker of treatment resistance. An imbalance between CD8^+^ early memory and senescent-like cells is associated with unfavorable treatment outcomes and low survival rates in AML patients. It was also postulated that, in contrast to solid tumors, BM T cell exhaustion is less prone to occur in AML, and CD8^+^ senescent-biased skewing is the principal mechanism of T cell dysfunction in AML [5]. This is the first study to elucidate the dynamic alterations in the differentiation and functional impairment of CD8^+^ T cells during AML treatment, facilitating advancements in the understanding of CD8^+^ T cell dysfunction in AML. In conclusion, T cell senescence is a relatively new area of research and does not yet have clinical applications in AML. At present, a heightened focus is being placed on its ramifications for disease progression and treatment outcomes. Nonetheless, there is a dearth of research on the mechanism of its interaction with AML. Hence, it is in its infancy and requires additional fundamental research to complement the roadmap of immune-based interventions.

### 3.2. Inducing Mechanism of T Cell Senescence in the AML Microenvironment

The bone marrow microenvironment containing AML blast cells is typically immunosuppressive, and the balance of immunity is crucial to the efficacy of immunotherapy and other treatments (such as chemotherapy and targeted therapy) [43,44]. In addition to the influence of diverse immunosuppressive cells and immunosuppressive factors, the functionality of T cells is impaired and gradually progresses toward exhaustion or senescence. Xin Wu et al. deciphered the remodeling of the immune microenvironment in AML through single-cell multidimensional analysis. The immune microenvironments of AML with complete remission (AML-CR) and AML with no response (AML-NR) are highly disparate, and the E3 ubiquitin ligase RNF149 alters the immune milieu of AML, triggering dysfunction in CD8^+^ T cells [45]. Previous investigations have demonstrated that senescence is the predominant cause of T cell dysfunction in AML [5]. Hence, a precise understanding of the mechanism of T cell senescence within the AML microenvironment would contribute to overcoming immune dysfunction. T cell senescence includes replicative and premature senescence. Replicative senescence occurs when cells cease division, as their telomeres progressively shorten after multiple divisions. Premature senescence alludes to senescent T cells via continuous antigenic stimulation, chronic inflammation, oxidative stress, and other stressors independent of telomeres [46,47]. Maurice Reimann et al. insist that senescence cannot be characterized as an entirely reversible or irreversible process. If the underlying molecular mechanisms of aging are not sustained, cells have the potential to re-enter the cell cycle. Unlike cells that have never undergone aging, rejuvenated senescent cells dynamically advance to postsenescent states featuring diverse functional attributes [48]. In organisms, reactive oxygen species (ROS) are predominantly generated via cellular metabolic procedures, and excessive production can result in oxidative stress and DNA damage, which constitute significant driving forces in numerous diseases and aging processes [49]. Senescent T cells rely more on anaerobic glycolysis than on the tricarboxylic acid cycle for energy production, thereby resulting in mitochondrial dysfunction and the accumulation of ROS [50]. The mitogen-activated protein kinase (MAPK) signaling pathway is crucial for T cell senescence and encompasses several prominent kinase families, among which the most prevalent are the extracellular signal-regulated kinase (ERK), c-Jun N-terminal kinase (JNK), and p38 families. Under the induction of diverse stress factors, the MAPK-related pathway is activated. Sestrins are the products of metabolic competition or oxidative stress in senescent cells, and they can inhibit TCR activation in *mice* and *humans*; influence the metabolism of T cells by regulating the mechanistic target of the rapamycin (mTOR) signaling pathway; and activate adenosine 5′monophosphate-activated protein kinase(AMPK) to upregulate P38, ERK, and JNK to facilitate aging [51]. Research indicates that the depletion of receptor-interacting protein kinase 1 (RIPK1) results in the increased basal activation of mTORC1, thereby inducing the premature senescence of T cells [52]. Additionally, senescent T cells fail to proliferate upon TCR stimulation, and the activities of signaling molecules such as lymphocyte-specific protein tyrosine kinase (Lck) and zeta-chain-associated protein kinase (ZAP70) might be diminished. The downregulation of TCR signaling can trigger the MAPK p38 signaling pathway or inhibit the PI3K-Akt-mTOR axis, subsequently leading to the inactivation of autophagy, the induction of mitochondrial dysfunction, and elevated ROS levels in senescent T cells [53]. Thus, mTOR may constitute a potential target for reversing T cell senescence.

Numerous prior studies have indicated that Tregs are particularly relevant to the occurrence and progression of T cell senescence. In AML, Tregs are frequently augmented and functionally enhanced within the tumor microenvironment and are correlated with an unfavorable prognosis [54,55,56]. The augmented competition for glucose between Treg cells and tumor cells triggers ATM-related DNA damage, which induces the ERK1/2 and p38 pathways, activates the STAT1/3, and upregulates *p53*-*p21*-*p16*, ultimately resulting in the stable withdrawal of T cells from the cell cycle [16,20,57]. Moreover, glucose energy deprivation is also capable of activating the AMPK pathway to downregulate the binding between the telomerase reverse transcriptase (TERT) gene and transforming growth factor-activated protein kinase 1 (TAB1), thereby giving rise to p38 self-phosphorylation and inducing T cell aging and endogenous DNA damage [58,59]. Lactic acid secreted by AML blast cells promotes the accumulation of programmed cell death protein-1(PD-1^+^) Tregs. Moreover, Treg cells convert CD4^+^ helper cells and CD8^+^ cytotoxic T cells into a dysfunctional senescent state [60]. ERK1/2 and p38 signaling cooperate with STAT1 and STAT3 to regulate the senescence of effector T cells induced by Tregs. Studies have indicated that vacuolar protein sorting 33B (Vps33B) governs the activation of amino acid signal-dependent mTORC1 and cell metabolism, thereby sustaining the suppressive function of Treg cells [61]. T cells maintain the balance of the immune response by expressing immune checkpoints (ICs) on their surface, preventing the occurrence of autoimmune reactions. To evade immune surveillance, leukemia stem cells strongly express aberrant IC ligands, such as programmed cell death ligand-1 (PD-L1) [62]. The PD-1/PD-L1 axis is capable of facilitating the proliferation of Tregs and the accumulation of senescent cells [63], thereby further hampering the effector functions of T cells [64]. Moreover, leukemia cells can also induce Tregs by releasing IFNγ, reshaping the bone marrow immune landscape [65].

**Figure 3:**
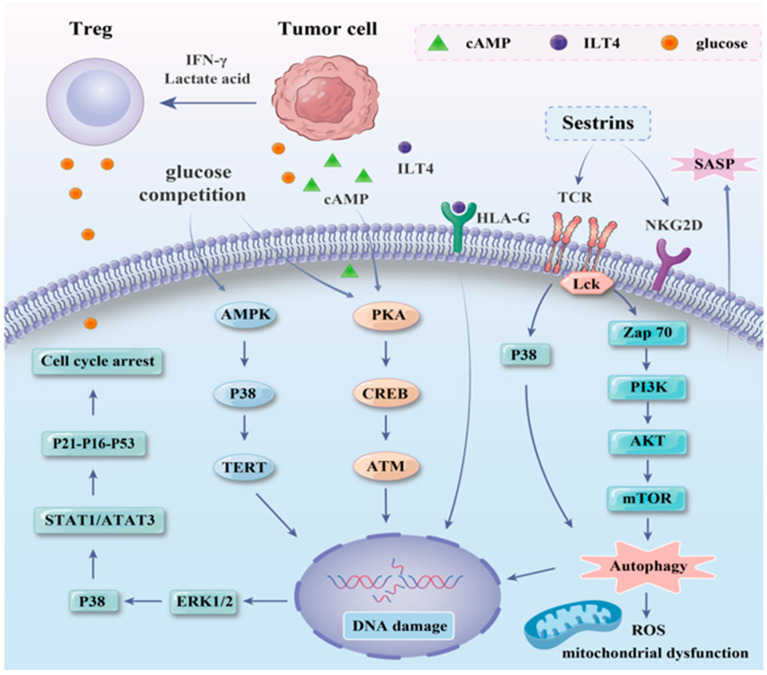
Key signaling pathways involved in T cell senescence within the tumor microenvironment. In the immunosuppressive microenvironment formed by tumor cells, the MAPK signaling pathway of T cells is persistently activated by the continuous stimulation of tumor antigens and metabolic competition. Glucose deprivation results in the phosphorylation of P38, resulting in telomere shortening, DNA damage, and cell cycle arrest. Additionally, TCR signal transduction in senescent T cells is downregulated, leading to autophagy inactivation and further exacerbating mitochondrial dysfunction. Treg: regulatory T cell, IFN-γ: interferon-gamma, cAMP: cyclic adenosine monophosphate, ILT4: immunoglobulin-like transcript 4, HLA-G: Human leukocyte antigen-G, PKA: phosphorylase kinase A, CREB: cAMP response element-binding protein, ATM: ataxia-telangiectasia mutated, AMPK: adenosine 5′monophosphate-activated protein kinase, TERT: telomerase reverse transcriptase, ERK: extracellular signal-regulated protein kinase, STAT: signal transducer and activator of transcription, LCK: lymphocyte-specific protein tyrosine kinase, ZAP70: zeta-chain-associated protein kinase 70, ROS: reactive oxygen species, SASP: senescence-associated secretory phenotype, TCR: T cell receptor, AKT: protein kinase B, PI3K: phosphatidylinositide 3-kinase, mTOR: mammalian target of rapamycin, NKG2D: natural killer group 2 member D.

Leukemia cells can also induce the transformation of T cells into senescent T cells by directly contacting and impairing the activation of T cells and inducing the generation of senescent markers through bystander regulation. The dysfunction of CD8^+^ T cells in AML might be attributed to T cell apoptosis, the activation of the IFN signaling pathway, and the inhibition of the costimulatory receptor (CD28, OX40, and ICOS) signaling pathways [8]. Notably, given the close proximity of leukemia blast cells to circulating T cells, their potential for facilitating T cell senescence could be more pronounced than that of solid tumors located at the periphery [7]. Cyclic adenosine monophosphate (cAMP) originates from tumors and is transferred to T cells via gap junctions. Likewise, cAMP is also a key component in the Treg-mediated suppression process [66] (Figure 3). The tumor-derived immunoglobulin-like transcript 4 (ILT4)/ILT4 ortholog in *mice* (PIR-B) activates ERK1/2 signal transduction to increase fatty acid synthesis and lipid accumulation in tumor cells and induce the senescence of effector T cells [67]. Similarly, exosomes have been verified to act as transporters for aging-related proteins. Ying Chen et al. demonstrated that the augmented expression of RAB27B in leukemia stem cells selectively facilitates the loading and release of exosomes enriched with aging-inducing proteins and induces the senescence of mesenchymal stem cells (MSCs) [68]. It can be deduced that exosomes within the AML microenvironment might also induce T cell senescence, thereby mediating immune evasion.

In summary, senescent T cells represent a particular state that breaks dormancy, resembles cell differentiation, and stems from the activation of DNA damage and programmed pathways associated with differentiation. The circulatory properties of hematological tumors determine their unique interactions with immune cells. Regrettably, most of the current mechanistic studies have been conducted on cell lines or in vitro experiments on solid tumors and have few specific molecular mechanisms in AML, which could be crucial for overcoming the dilemma of immunotherapy.

### 3.3. Functional Alterations in Senescent T Cells in the AML Microenvironment

A recent investigation discerned four CD8^+^ T cell subsets in AML patients via multimodal analysis: naïve, early T memory (early Tm), activated (act), and terminally differentiated/senescent-like (term/sen L) [5]. Both the senescence and exhaustion of T cells are two major dysfunctional states in AML patients. Under the pressure of long-term exposure to chronic antigenic stimulation and persistent immune activation, exhausted T cells highly express inhibitory receptors such as PD-1, T cell immunoglobulin and mucin-domain-containing-3 (TIM-3), cytotoxic T-lymphocyte antigen 4 (CTLA-4), and lymphocyte activation gene-3 (LAG-3). Moreover, the production of the main proinflammatory and cytotoxic cytokines, such as IL-2, TNF-α, and IFN-γ, is markedly reduced. Hence, they can be characterized as effector T cells with diminished cytokine expression and effector function [69,70,71,72]. Exhausted T cells are heterogeneous and represent a dynamic and progressive state, encompassing progenitor and terminal subpopulations with distinctive characteristics and responses to checkpoint blockade [73,74]. To enhance the understanding of T cell differentiation and functional disorders, Josephine et al. constructed the epigenetic and transcriptional maps of T cell differentiation from healthy individuals, including exhausted CD8^+^ T cells [75]. Unlike exhausted T cells, senescent T cells possess a markedly impaired capacity for lysing leukemia cells, irreversible cell cycle arrest, and a deficiency in proliferative capacity. However, they still secrete copious amounts of proinflammatory cytokines, except for IL-2 [76]. This chronic inflammatory stimulation is capable of facilitating the proliferation and survival of AML cells. In functional research, CD8^+^CD57^+^ T cells derived from AML patients manifested irreversible proliferation arrest that could not be rescued by IL-2 [8]. Upon stimulation with anti-CD3 and anti-CD28 antibodies, the quantities of IL-2 and IFN-γ generated by senescent T cells are markedly lower than those generated by other T cells [77]. Interestingly, despite the compromised TCR signal transduction of senescent T cells, their innate-like functions are augmented, as manifested by the upregulation of natural killer cell receptors (NKRs) [78,79]. Senescent T cells possess effective inhibitory activity, which is not accomplished through the inhibition of the cytokines IL-10 and TGF-β [16]. T cells and certain inhibitory T cells exhibit similar phenotypes, indicating that they might constitute the same population, namely, a particular form of Tregs. In brief, senescent T cells are a group of cells with strong immune-suppressive effects. Accurately discriminating the functional phenotypes of T cells in AML is crucial to tailor the appropriate treatment for individuals.

## 4. Intervention Strategies for T Cell Senescence in the AML Microenvironment

Despite the promising clinical responses that have been observed in some patients with blood malignancies treated with T cell-targeted immunotherapy, others do not exhibit sustained clinical responses or endure intolerant immune-related adverse events. Senescent T cells assist in the evasion of immune surveillance and the clearance of malignant tumors. Hence, controlling and reversing the senescent status of T cells is of paramount importance for antitumor immunity.

### 4.1. Blocking the Key Signaling Pathway of T Cell Senescence

The pivotal target for reversing T cell senescence is P38MAPK. Many preclinical experiments have demonstrated that inhibitors targeting the p38, ERK, JNK, and STAT signaling pathways can potentially prevent T cell senescence [80]. Inhibitors targeting p38MAPK restore telomerase activity and alleviate cell cycle arrest during the aging process in T cells by blocking key pathways [81]. Research findings have indicated that the combination of ATM and/or MAPK signaling inhibition and anti-PD-L1 checkpoint blockade can synergistically augment antitumor immune efficacy in vivo [82]. Compared with that of AMPK, the inhibition of the P38 pathway is more selective. Its safety has been verified in other hematological malignancies, and it has been extensively applied in the treatment of melanoma patients [83,84]. AMPK acts as a central regulator, featuring extensive connections and low specificity, rendering it inapplicable for therapeutic purposes. Notably, cell aging and cell immortality are mutually convertible, and inhibiting the aging process of cells has the potential to promote cancer development. Hence, the safety and efficacy of MAPK inhibitors still require extensive clinical trials and time for verification.

### 4.2. Metabolic Regulation Targeting T Cell Senescence

Mitochondrial dysfunction and metabolic disorders might manifest internal dysregulation within senescent T cells induced by external factors [85]. Toll-like receptor 8 (TLR8), rapamycin, and metformin can target metabolism to facilitate the restoration of T cell activity. A growing body of evidence indicates that TLRs directly govern metabolism and can also directly modulate energy metabolism in immune cells, influencing the tumor behavior and function of melanoma, prostate cancer, head and neck cancer, and breast cancer [86]. TLR8 signal transduction within human Treg cells impedes their induction of senescence in effector T cells and dendritic cells (DCs) and is able to reverse the inhibitory function of senescent T cells [87]. The addition of glucose prevents Treg-induced effector T cells from senescing during the interaction process [88]. TLR8 agonists diminish the generation of cAMP in tumor cells and restrain glycolysis in tumor-derived Tregs without interfering with the metabolism of effector T cells, thereby preventing T cell senescence. Likewise, cAMP inhibitors (7-ddA), or synthetic poly-G3 and natural TLR8 ligands (ssRNA40), can also suppress tumor- and Treg-induced T cell aging by reactivating TLR8 signaling [10,20]. Motolimod, a TLR8 agonist, can also treat AML by inducing inflammatory cell death, exerting negligible effects on normal lymphocytes [89]. Currently, numerous clinical trials of TLR agonists are ongoing since TLRs constitute potential targets for the treatment of AML relapse and drug resistance [90,91]. mTOR plays a central role in modulating numerous fundamental cellular processes, ranging from protein synthesis to autophagy. The mTOR pathway is among the pathways that are evidently associated with senescence and can suppress the secretory phenotype of senescent cells. mTOR inhibitors might retard and reverse cell aging [92,93,94,95]. Research has shown that cyclooxygenase-2 (COX-2)/soluble epoxide hydrolase (sEH) inhibitors can restore autophagy via the mTOR pathway and mitigate cellular aging in *mice* [53]. Furthermore, metformin, a first-line medication for diabetes treatment, also has the capacity to increase autophagy and normalize mitochondrial function [96,97]. Further studies revealed that metformin is capable of systematically ameliorating the senescent traits of multiple tissues and organs throughout the aging body, including epigenetic alterations, oxidative damage, the accumulation of senescent cells, the infiltration of immune cells, and the establishment of an inflammatory milieu, and thus can be employed as a tool for targeting cell aging [98]. Liu et al. proposed that senescent T cells driven by malignant tumor cells and regulatory T cells exhibit imbalanced lipid metabolism. Inhibiting lipid metabolism in effector T cells through reprogramming with group IVA phospholipase A2 (PLA2G4A) has a favorable effect on preventing ex vivo T cell senescence, which has been validated in mouse models of melanoma and breast cancer [99]. In summary, alterations in T cell metabolism might serve as crucial targets for regulating T cell dysfunction and could be combined with other therapeutics in the future to enhance the efficacy of immunotherapy.

### 4.3. Cellular Immunotherapy Targeting T Cell Senescence

Adoptive cell immunotherapy (ACT) represents a cancer therapeutic approach that enhances a patient’s antitumor immune response through in vitro expansion, activation, or genetic modification of the patient’s own or donor immune cells, followed by reinfusion into the patient [100]. ACT includes several principal forms, such as chimeric antigen receptor (CAR)-T cell therapy, T cell receptor-engineered T (TCR-T) cell therapy, and NK cell therapy, the efficacy of which is highly reliant on functionally normal and active T cells and is emerging as a potentially curative treatment for patients with advanced hematological malignancies [101,102,103]. CAR-T cells generated by dysfunctional T cells exhibit inferior therapeutic efficacy in targeting leukemia and other hematological malignancies [104,105]. There are three strategies to address the dormancy of T cell pools: (1) select and optimize T cell populations, identify markers of T cell senescence, and induce apoptosis [106] or the selective depletion of terminally differentiated populations [107]. To prioritize gene and protein candidates that have the potential to identify and eliminate senescent cells selectively, the SenoRanger gene set offers a dependable hypothesis regarding potential valuable targets for the identification and elimination of senescent cells [108]. (2) By using CRISPR/Cas9 gene editing to knock out or suppress negative regulatory genes that inhibit the differentiation or activation of immune cells, the persistence and efficacy of CAR-T/CAR-NK cells in killing tumors can be enhanced [109]. (3) In vitro amplification and cultivation techniques should be enhanced. In the expansion culture using an optimized cytokine mixture, cytotoxic T lymphocytes (CTLs) can proliferate abundantly even in the absence of a feeder layer [110]. Moreover, γ chain cytokine therapy can ameliorate senescent CAR-T cells [111], mainly by activating the Janus kinase (JAK)–STAT signaling pathway [112]. For example, IL-7-producing immunotherapy improves the ex vivo T cell functions of immunosenescent patients [113], and IL-7 can sustain the CD27/CD28 expression and proliferative capacity to restrain tumor-induced T cell senescence [114]. Similarly, IL-15 can stimulate tumor antigen-specific T cell expansion and effector function [93]. In addition, type 2 cytokine signaling protects macrophages from immune aging by activating the IL-4-STAT6 pathway [115]. NK cells are innate cytotoxic lymphocytes involved in monitoring and eliminating cancer. Studies have demonstrated that autologous NK cell infusion can significantly alleviate T cell senescence and exhaustion and serves as a practical approach to suppress the key components of the SASP. After NK cell infusion, the quantities of CD28^−^, CD57^+^, CD28^−^CD57^+^, and CD28^−^KLRG1^+^ CD4^+^ and CD8^+^ T cells, which represent aging cells, decrease significantly [116]. Another clinical study demonstrated that, upon the infusion of autologous NK cells in patients, the levels of aging markers decreased, and aging-related inflammatory responses were inhibited [117]. Nevertheless, the mechanism by which NK cells influence T cell senescence remains elusive.

## 5. Discussion

Miriam Merad et al. presented the first compelling evidence demonstrating that chronic inflammation induced by an aging immune system is likely to result in cancer [118]. Senescence and cancer might therefore be mutually causal. Over the past several years, considerable advancements have been made in comprehending and probing the correlation between senescent T cells and the clinical prognosis of AML patients. Immune testing of blood or bone marrow samples could be applied to predict treatment response, high risk for relapse, and adverse prognosis. Immune gene expression profiles and IED scores are capable of enhancing AML risk stratification. It is better to integrate IED scores and basic genomics to assess the individual survival of AML patients more precisely. The immune status can be instructive for clinical decisions.

At present, a high-dimensional analysis of the TME in AML is lacking. In the era of rapid computer advancement, progress in information sharing and experimental techniques will contribute to uncovering how the dynamic evolution of the AML microenvironment influences the phenotypic transformation of T cells. Moreover, more evidence is needed to elucidate the dynamic transformation mechanism of various T cells during the immune editing process [119]. AML is a complex disease with heterogeneity in the microenvironment, which poses a challenge in comprehending the influence of diverse cell types on disease progression [120]. The impacts of diverse AML subtypes and different degrees of LSCs on T cell dysfunction are likely to be distinct, and the immune composition of the microenvironment in every patient remains unique. If we can reveal the influence of AML heterogeneity on T cell differentiation by establishing cell-specific biomarkers at each stage, it will significantly promote the advancement of immunotherapy and precision medicine. This demands the collection of substantial amounts of patient data and longitudinal specimens. Moreover, a more specific and in-depth exploration of T cell senescence mechanisms is needed. Finally, researchers have not determined whether leukemia stem cells, the “hidden killers” in the AML microenvironment, are associated with the induction of T cell senescence, which plays a dual role in the treatment of AML; it can be said to kill two birds with one stone.
